# Unintegrated HIV-1 provides an inducible and functional reservoir in untreated and highly active antiretroviral therapy-treated patients

**DOI:** 10.1186/1742-4690-4-60

**Published:** 2007-08-29

**Authors:** Gaël Petitjean, Yassine Al Tabaa, Edouard Tuaillon, Clement Mettling, Vincent Baillat, Jacques Reynes, Michel Segondy, Jean Pierre Vendrell

**Affiliations:** 1 Laboratoire de Virologie, Hôpital Lapeyronie, Avenue du Doyen Gaston Giraud, 34295 Montpellier, France; 2 Unité INSERM 847, France; 3 Université Montpellier 1, Boulevard Henri IV, 34967 Montpellier Cedex 2, France; 4 Institut de Génétique Humaine, Centre National de la Recherche Scientifique, Unité Propre de Recherche 1142, Montpellier, France; 5 Département des Maladies Infectieuses et Tropicales, Hôpital Gui de Chauliac, Avenue Bertin Sans, 34295 Montpellier, France; 6 Laboratoire de Virologie, Hôpital Saint Eloi, 80 Avenue Augustin Fliche, 34295 Montpellier, France

## Abstract

**Background:**

The presence of HIV-1 preintegration reservoir was assessed in an *in vitro *experimental model of latent HIV-1 infection, and in patients treated or not with highly active antiretroviral therapy (HAART).

**Results:**

In resting CD4^+ ^T lymphocytes latently infected *in vitro *with HIV-1, we demonstrated that the polyclonal activation induced a HIV-1 replication, which could be prevented by the use of an HIV-1 integrase inhibitor. We also showed that this reservoir was labile since the rescuable HIV-1-antigens production from unintegrated HIV-1 genomes declined over time. These data confirm that our experimental approach allows the characterization of a functional unintegrated HIV-1 reservoir. We then explored the preintegration reservoir in HIV-1-infected patients. This reservoir was detected in 11 of 12 untreated patients, in 4 of 10 sustained responders to HAART, and in one incomplete responder. This reservoir was also inducible, labile, and anti-HIV-1 integrase drug inhibited its induction. Finally, this reservoir was associated with the presence of spontaneous HIV-1 antigens producing CD4^+ ^T cells in blood from 3 of 3 untreated patients and 2 of 2 sustained responders to HAART harboring a preintegration reservoir.

**Conclusion:**

This preintegration phase of HIV-1 latency could be a consequence of the ongoing viral replication in untreated patients and of a residual viral replication in treated patients.

## Background

In human immunodeficiency virus type 1 (HIV-1)-infected patients, replication-competent virus persists in a long-lived reservoir comprised of resting CD4^+ ^T lymphocytes latently infected with HIV-1. These cells appear when productively infected CD4^+ ^T lymphoblasts escape from both immune response and cytopathic effects of the virus and revert to a resting memory state [1]. Memory CD4^+ ^T cells that have integrated HIV-1 DNA in their genome characterize the postintegration phase of latency [2]. Infected CD4^+ ^T cells harboring unintegrated HIV-1 DNA, which constitute a second form of latency named preintegration latency, are observed immediately after direct infection of resting CD4^+ ^T cells [2]. In these cells, post-entry blocks in virus life cycle result from the inability to complete reverse transcription or failure to import the preintegration complex into the nucleus. This could be due to insufficient levels of nucleotide precursors and stores of ATP required for the PIC translocation [3] and entry into the cell cycle [4,5]. However, these blocks can be surmounted through activation of infected resting CD4^+ ^T lymphocytes [2,6-8].

In HIV-1-infected individuals, the presence of unintegrated viral genome in resting CD4^+ ^T lymphocytes is sustained by the fact that latently HIV-1-infected resting CD4^+ ^T cells during the follow-up of acute seroconverters treated early with highly active antiretroviral therapy (HAART) shows a biphasic decay [9-11]. After an initial fast decay, HIV-1-infected resting CD4^+ ^T cells declines at a slower rate, reflecting the turnover of a longer-lived viral reservoir in infected cell population. The two phases of this decay are related to the two different forms of latency and support models of pre- and postintegration latency [10]. In untreated patients, there is an active viral replication with continual infection of resting T cells, leading to a labile pool of cells in the preintegration phase of latency. When HAART is initiated, viral replication ceases, probably leading to the rapid decay of this labile reservoir [9,12-15]. However, the persistence of preintegrated forms of HIV-1 could be explained by the *de novo *infection of resting CD4^+ ^T cells due to residual viral replication [15-18][19].

All data available on the preintegration state result from molecular studies in untreated patients [12] or from *in vitro *infection model of resting CD4^+ ^T cells [7,15]. Nevertheless, the functional unintegrated HIV-1 reservoir, able to generate rescuable virus production, has not been observed in sustained responders to HAART. In previous studies, we developed an HIV-1-antigen-ELISpot assay (HIV-1-Ag-ELISpot) for the enumeration of HIV-1-antigen-secreting cells (HIV-1-Ag-SCs) after *in vitro *polyclonal activation of highly purified resting CD4^+ ^T lymphocytes [20-22]. We reported that the CD4^+ ^T cell stimulation induced a higher number of HIV-1-Ag-SCs in untreated patients comparatively with HAART-treated patients [21]. Thus, we hypothesized that this discrepancy could be explained by the presence of unintegrated viral genomes able to enter a replicative cycle in stimulated CD4^+ ^T lymphocytes from untreated patients. In this study, we assessed the capacity of the preintegration reservoir to produce rescuable HIV-1-antigens from resting CD4^+ ^T cells after polyclonal activation in an *in vitro *model of HIV-1 latent infection of resting CD4^+ ^T lymphocytes. We then observed that unintegrated viral reservoir could provide an inducible and functional reservoir for HIV-1 in untreated patients as well as in patients with sustained response to HAART.

## Results

### Characterization of the preintegration reservoir in an in vitro model of HIV-1 infected CD4^+ ^T lymphocytes

**Figure 1:**
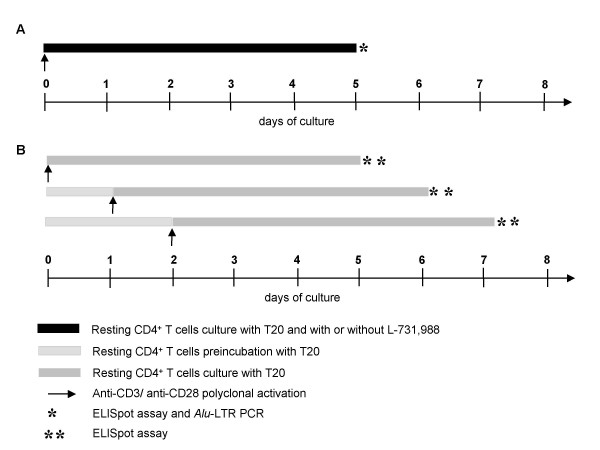
**Experimental protocol and culture conditions**. **A**. In order to study the mobilization of the functional preintegration reservoir, resting CD4^+ ^T cells were activated and cultured with the HIV-1 integrase inhibitor L-731,988 at the final concentration of 40 μM. **B**. To assess the correlation between the unintegrated HIV-1 DNA decay *in vitro *and the decline of rescuable viral production, infected resting CD4^+^T cells were preincubated 1 or 2 days before polyclonal stimulation. In both cases, in order to prevent infection of others cells by *de novo*-synthesized HIV-1, 1 μg/ml of the viral entry inhibitor T20 was also added in culture medium.

**Figure 2:**
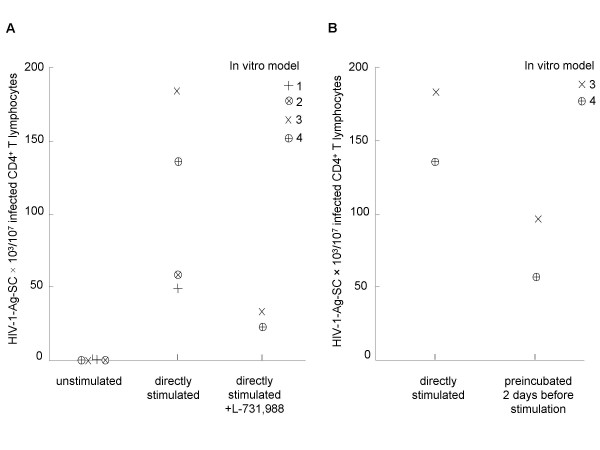
**In vitro model of latently infected resting CD4^+ ^T cells**. **A**. The experimental approach was validated using *in vitro *latently infected resting CD4^+ ^T cells that were unstimulated and directly polyclonaly activated in four experiments (nos. 1, 2, 3, and 4) or directly polyclonaly activated and cultured with L-731,988 in two other assays (nos. 3 and 4). **B**. *In vitro *latently infected resting CD4^+ ^T cells were directly polyclonaly activated or preincubated 2 days before polyclonal activation in two experiments (nos. 3 and 4).

*In vitro *latently infected resting CD4^+ ^T cells obtained with the experimental protocol of infection were tested by ELISpot to enumerate replication-competent infected cells before and after polyclonal activation (Fig. 1A). Cells were cultured with T20 to avoided *de novo *infections. In four (nos. 1, 2, 3, and 4) polyclonal T cell activation experiments (Fig. 2A), 49,200 to 184,000 HIV-1-Ag-SCs/10^7 ^resting CD4^+ ^T lymphocytes were enumerated (mean, 106,435 HIV-1-Ag-SCs/10^7 ^resting CD4^+ ^T lymphocytes), whereas unstimulated infected cells generated only <1 to 100 HIV-1-Ag-SCs/10^7 ^resting CD4^+ ^T cells (mean, 35 HIV-1-Ag-SCs/10^7 ^resting CD4^+ ^T lymphocytes). To address the presence of a functional preintegration HIV-1 reservoir, infected resting CD4^+ ^T lymphocytes were stimulated and cultured with or without addition of the HIV-1 integrase inhibitor L-731,988. In two experiments (nos. 3, 4), we enumerated 135,740 and 184,000 HIV-1-Ag-SCs/10^7 ^resting CD4^+ ^T cells. In contrast, only 22,900 and 33,620 HIV-1-Ag-SCs/10^7 ^resting CD4^+ ^T cells were enumerated when cells were cultured with L-731,988 (Fig. 2A). These results suggest that the *in vitro *polyclonal activation of resting CD4^+ ^T lymphocytes induces the integration of some extrachromosomal HIV-1 genomes as previously described in other reports [12,13,23] and clearly demonstrates that our method allows for the detection of an inducible functional preintegrated HIV-1 reservoir.

### Impact of unintegrated HIV-1 DNA decay on the functional preintegration reservoir

*In vitro *infected resting CD4^+ ^T lymphocytes were preincubated or not for 2 days before cell polyclonal activation (Fig. 1B). After 5 days of culture, cells were tested by ELISpot assay. Cells were cultured with T20. In two experiments (nos. 3, 4), we enumerated 184,000 and 135,700 HIV-1-Ag-SCs/10^7 ^resting CD4^+ ^T lymphocytes in the absence of preincubation, and only 97,000 and 57,000 HIV-1-Ag-SCs/10^7 ^preincubated resting CD4^+ ^T cells (Fig. 2B). It was thus observed a decrease in the rescuable viral production from preincubated latently infected cells and these results are in agreement with other molecular studies demonstrating that unintegrated HIV-1 DNA is unstable *in vitro *[15,23].

### Functional preintegration reservoir in untreated patients

**Figure 3:**
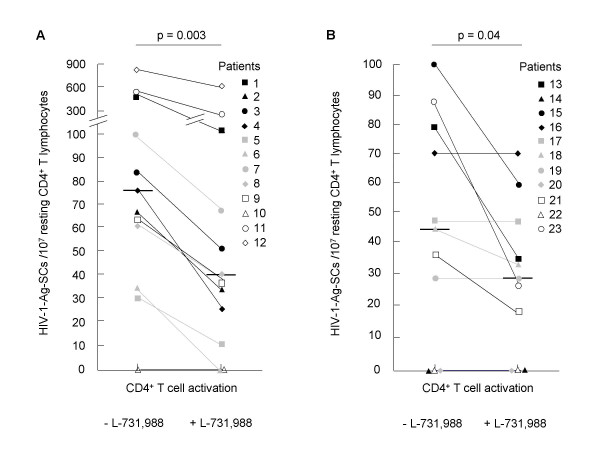
**Mobilization of the functional preintegration reservoir. Resting CD4^+ ^T lymphocytes secreting HIV-1 viral proteins in untreated (A) and HAART-treated patients (B)**. The CD4^+ ^T lymphocytes were polyclonally activated and cultured with 1 μg/ml of enfuvirtide and with or without 40 μM of the HIV-1 integrase inhibitor L-731,988. The median values are shown as black bars. Comparison of results was done by the Wilcoxon signed-rank test.

To detect the functional preintegration reservoir in HIV-1-infected patients, resting CD4^+ ^T lymphocytes were isolated and purified from blood samples from 12 untreated patients (Fig. 3A). To determine the fraction of resting CD4^+ ^T cells carrying functional HIV-1 preintegration reservoir, cells were polyclonally activated and cultured with or without L-731,988. Cells were cultured with T20. The HIV-1 reservoir was detected in 11/12 untreated patients (91.6%). HIV-1-Ag-SCs were not detected for patient no. 10 and this observation was explained by clinical data indicating a long-term non-progressor state characterized by undetectable plasma viral load and steady-state high CD4^+ ^T cell count (Table 1). For the 11 other patients, HIV-1-Ag-SCs induced by polyclonal activation of resting CD4^+ ^T lymphocytes ranged from 28.57 to 825 HIV-1-Ag-SCs/10^7 ^resting CD4^+ ^T cells (median, 75 HIV-1-Ag-SCs/10^7 ^resting CD4^+ ^T cells; 25^th^–75^th ^percentiles, 61.25–291.66 HIV-1-Ag-SCs/10^7 ^resting CD4^+ ^T cells). When resting CD4^+ ^T cells were activated and cultured with L-731,988, we observed a significant decrease (*P *= 0.003) in HIV-1-producing cells since <1 to 675 HIV-1-Ag-SCs/10^7 ^resting CD4^+ ^T lymphocytes (median, 40 HIV-1-Ag-SCs/10^7 ^resting CD4^+ ^T cells; 25^th^–75^th ^percentiles, 29.16–102.77 HIV-1-Ag-SCs/10^7 ^resting CD4^+ ^T cells) were enumerated. For one seronegative patient with primary HIV-1 infection (no. 6), rescuable antigen-producing cells were not detected when resting CD4^+ ^T lymphocytes were cultured with the integrase inhibitor and this result suggests that only a functional preintegration reservoir was detectable at the time of sampling. The preintegration reservoir was thus detected in 100% of untreated patients with detectable plasma viral load.

**Table 1:** Characteristics of the HIV-1-infected patients studied.

Patients	Drug regimen at the time of the study	Duration of virologic suppression (month)	Plasma HIV-1 RNA level (copies/ml)	CD4^+ ^T cell count (cells/μl)
1	naive		5,634	264
2	naive		21,580	291
3	naive		1,246	460
4	naive		72,539	315
5	naive		42,536	574
6	naive		2,156,097	656
7	pti		184,713	271
8	pti		163,435	406
9	pti		11,869	697
10	naive		<50	945
11	pti		2,276	493
12	npti		40,000	322
13	3TC+ABC+NVP		1,140	998
14	3TC+ABC+NVP	48	<50	448
15	ABC+3TC+NFV	34	<50	185
16	ABC+3TC+NVP	60	<50	460
17	3TC+EFV+TNV	39	<50	892
18	AZT+ABC+3TC+LVP/RTV	1	<50	479
19	3TC+TNV+SQV/RTV	1	<50	1,151
20	3TC+TNV+NVP	67	<50	468
21	TNV+ABC	39	<50	527
22	3TC+ABC+LVP/RTV	19	<50	63
23	AZT+3TC+DDI	68	<50	995

pti, programmed treatment interruption; npti, non-programmed treatment interruption.

### Functional preintegration reservoir in HAART-treated patients

We then explored the functional preintegration reservoir in 10 sustained responders and one incomplete responder to HAART (Fig. 3B). Functional HIV-1 reservoir was not detected in 3/11 (27.3%) HAART-treated patients (nos. 14, 20, and 22); this observation could be explained by the fact that the frequency of replication-competent resting CD4^+ ^T lymphocytes was less than 1 HIV-Ag-SCs/10^7 ^resting CD4^+ ^T cells. For the 8 other patients, resting CD4^+ ^T cells generated 28.57 to 100 HIV-1-Ag-SCs/10^7 ^resting CD4^+ ^T cells (median, 58.33 HIV-1-Ag-SCs/10^7 ^resting CD4^+ ^T cells; 25^th^–75^th ^percentiles, 42.42–80.80 HIV-1-Ag-SCs/10^7 ^resting CD4^+ ^T cells) after polyclonal activation. The addition of L-731,988 in culture medium significantly modified (*P *= 0.04) the number of replication-competent infected cells that generated 18.18 to 70 HIV-1-Ag-SCs/10^7 ^resting CD4^+ ^T lymphocytes (median, 34.52 HIV-1-Ag-SCs/10^7 ^resting CD4^+ ^T cells; 25^th^–75^th ^percentiles, 28–49.99 HIV-1-Ag-SCs/10^7 ^resting CD4^+ ^T cells). In three patients (nos. 16, 17, and 19), the functional HIV-1 reservoir was not modified by addition of the integrase inhibitor. Five patients including four sustained responders and one incomplete responder (nos. 15, 18, 21, 23, and 13, respectively), harbored a functional preintegrated reservoir.

### Lability of the functional preintegration reservoir in patients

**Figure 4:**
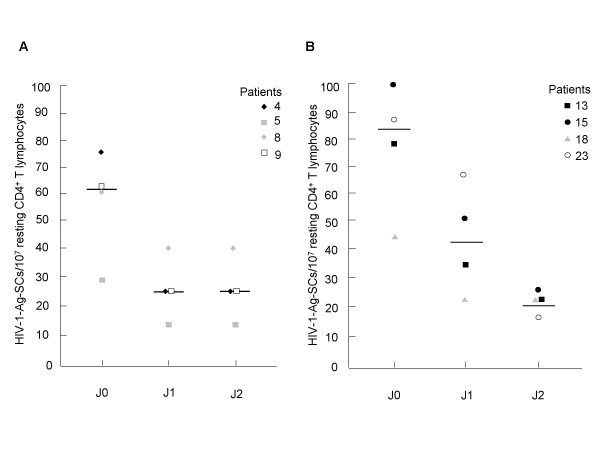
**Functional preintegration reservoir decay over the time in untreated patients (A) and in HAART-treated patients harboring a functional preintegration reservoir (B)**. Resting CD4^+ ^T lymphocytes were polyclonally activated (J0) or preincubated 1 (J1) and 2 days (J2) before stimulation. HIV-1-Ag-SCs were enumerated at the end of culture. The median values are shown as black bars.

Purified resting CD4^+ ^T cells from 8 of 16 HIV-1-infected patients harboring a functional preintegration reservoir were preincubated or not before their polyclonal activation and then tested by ELISpot assay. As shown on Fig. 4A, for four untreated patients (nos. 4, 5, 8, and 9) the number of HIV-1-Ag-SCs obtained after 1 or 2 days of preincubation decreased comparatively to HIV-1-Ag-SCs generated from CD4^+ ^T cells that were not preincubated. Indeed, HIV-1-Ag-SCs ranged from 28.57 to 75 HIV-1-Ag-SCs/10^7 ^resting CD4^+ ^T cells without preincubation (median, 61.25 HIV-1-Ag-SCs/10^7 ^resting CD4^+ ^T cells), from 14.28 to 40 HIV-1-Ag-SCs/10^7 ^resting CD4^+ ^T cells with one-day preincubation (median, 25 HIV-1-Ag-SCs/10^7 ^resting CD4^+ ^T cells), and from 14.28 to 40 HIV-1-Ag-SCs/10^7 ^resting CD4^+ ^T cells with two-days preincubation (median, 25 HIV-1-Ag-SCs/10^7 ^resting CD4^+ ^T cells).

For three sustained responder patients (nos. 15, 18, and 23) and one incomplete responder (no. 13), as shown on Fig. 4B, HIV-1-Ag-SCs ranged from 44.44 to 100 HIV-1-Ag-SCs/10^7 ^resting CD4^+ ^T cells without preincubation (median, 83.03 HIV-1-Ag-SCs/10^7 ^resting CD4^+ ^T cells), from 22.22 to 68.42 HIV-1-Ag-SCs/10^7 ^resting CD4^+ ^T cells with one-day preincubation (median, 41.66 HIV-1-Ag-SCs/10^7 ^resting CD4^+ ^T cells), and from 21.42 to 25 HIV-1-Ag-SCs/10^7 ^resting CD4^+ ^T cells (median, 21.82 HIV-1-Ag-SCs/10^7 ^resting CD4^+ ^T cells) with two-days preincubation. These results showed a decrease of rescuable viral production at day 1 and day 2. Thus, the inducible unintegrated HIV-1 DNA reservoir is unstable *in vitro *andthis observation is in agreement with the results of our *in vitro *experimental model of latent HIV-1 infection.

**Figure 5:**
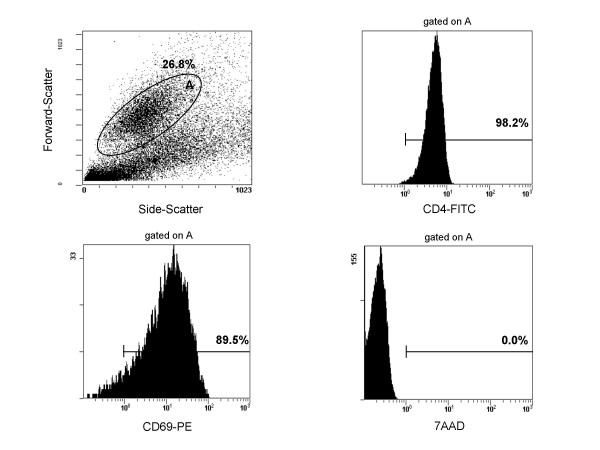
**Safeguarding of CD4^+ ^lymphocytes viability**. Representative flow cytometry histograms (patient no. 9) characterizing viability of CD4^+ ^T cell subset at the end of culture when cells were preincubated one or two days before their polyclonal activation. A gate **A **was set on the forward-scatter vs side-scatter histogram. As shown on different histograms, gate **A **corresponded to CD69^+ ^CD4^+ ^T lymphocytes. The analysis of the 7AAD level expression demonstrated that activated CD4^+ ^T lymphocytes were viable cells.

We then assessed if the decay of the number of HIV-1-Ag-SCs generated after one- and two-days preincubation was due to cell death. CD4^+ ^T cells viability was analyzed by flow cytometry after two days of preincubation and five days of culture. For patients nos. 8, 9, 15, and 23, cell viability analysis using the 7AAD marker showed that 86.1 to 100% CD4^+ ^T lymphocytes (median, 95.15%) were negative for 7AAD labelling and were considered as viable cells (Fig. 5). These results suggested that the decline of HIV-1-Ag-SCs could not be related to cellular death.

### Spontaneous HIV-1-producing CD4^+ ^T lymphocytes in patients

We finally assessed the number of *ex vivo *spontaneous HIV-1-Ag-secreting CD4^+ ^T lymphocytes in blood samples from three untreated patients (nos. 8, 11, 12) and from four sustained responders to HAART (nos. 15, 19, 21, and 22). For this purpose, freshly purified CD4^+ ^T lymphocytes not depleted of activated cells, were directly tested by ELISpot assay without activation stimuli and cultured with T20. (Table 2). Spontaneously HIV-1-Ag-secreting CD4^+ ^T lymphocytes were detected in 3/3 untreated patients (nos. 8, 11, 12) and in 2/4 sustained responders to HAART (nos. 15 and 21). In these 7 patients, infected cells showing spontaneous HIV-1 replication were present in 5/5 patients harboring a preintegration reservoir (nos. 8, 11, 12, 15, and 21) but were not observed in the 2 other patients without detectable preintegration reservoir (nos. 19, 22). These results highlight the fact that unintegrated HIV-1 reservoir could result from ongoing viral replication in patients with undetectable or low plasma viremia.

**Table 2:** Spontaneous HIV-1-antigen-producing CD4^+^T lymphocytes in HAART-treated and untreated patients.

Patients	Preintegration HIV-1 reservoir	*Ex vivo *HIV-1-Ag-SCs/10^7 ^CD4^+ ^T lymphocytes
8^a^	+^c^	26
11^a^	+	15
12^a^	+	30
15^b^	+	30
19^b^	-^d^	<1
21^b^	+	20
22^b^	-	<1

^a^Untreated patients.

^b^Sustained responder to HAART.

^c^Detection of functional preintegration reservoir.

^d^No detection of functional preintegration reservoir.

### Characterization of preintegrated HIV-1 DNA using Alu-LTR real-time PCR in the model of latent infection and in infected patients

**Figure 6:**
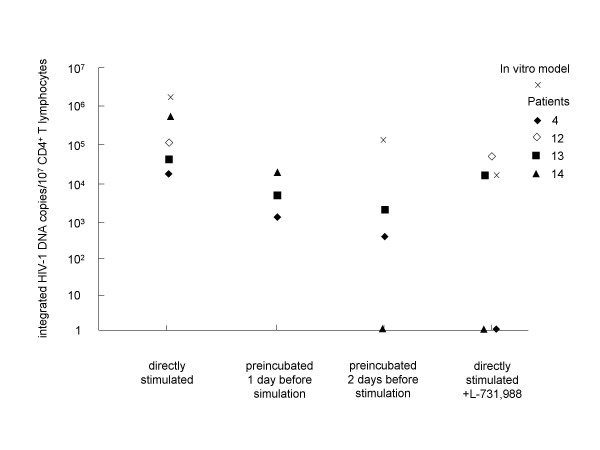
**Characterization of preintegrated HIV-1 DNA using *Alu*-LTR real-time PCR**. The level of integrated HIV-1 DNA copies was assessed in CD4^+ ^T lymphocytes from the *in vitro *model of infection and from four patients (nos. 4, 12, 13 and 14) using *Alu*-LTR real-time PCR experiments. CD4^+ ^T cells that were directly stimulated, preincubated 1 and 2 days before polyclonal activation, and directly stimulated and cultured with L-731,988 were recovered from ELISpot assays and tested in PCR experiments.

In the model of latent infection as well as in two untreated patients (nos. 4, 12), three sustained responder to HAART (nos. 14, 15, 16) and one incomplete responder (no. 13), polyclonally activated CD4^+ ^T lymphocytes cultured with or without integrase inhibitor were recovered after ELISpot assay to quantify the level of integrated HIV-1 DNA by PCR (Fig. 6). Cells were cultured with T20 to avoided *de novo *infections. In the *in vitro *infection model, integrated HIV-1 DNA levels were 1,873,330 copies/10^7 ^resting CD4^+ ^T cells, and 16,600 copies/10^7 ^resting CD4^+ ^T cells cultured with L-731,988. For two untreated patients, we detected 16,600 and 113,100 integrated HIV-1 DNA copies/10^7 ^resting CD4^+ ^T lymphocytes. However, <100 and 68,300 HIV-1 integrated DNA copies/10^7 ^resting CD4^+ ^T cells cultured with L-731,988, were respectively quantified. In one sustained responder (no. 14), integrated HIV-1 DNA level was 585,200/10^7 ^resting CD4^+ ^T lymphocytes, whereas we did not detect integrated HIV-1 DNA in resting CD4^+ ^T cells cultured with L-731,988. In two other sustained responders to HAART (nos. 15, 16), signals generated by integrated HIV-1 DNA were too weak to efficiently quantify HIV-1 proviruses. Finally, for the incomplete responder, we quantified 44,200 HIV-1 integrated DNA copies/10^7 ^resting CD4^+ ^T cells and only 18,000 HIV-1 integrated DNA copies/10^7 ^resting CD4^+ ^T cells cultured with L-731,988. Thus, the addition of integrase inhibitor decreased the number of integrated HIV-1 DNA copies and explained the decrease observed in the number HIV-1-Ag-SCs (Fig. 2A and 2B).

We then assessed the decay of unintegrated HIV-1 DNA in cells that were preincubated for one and two days before stimulation (Fig. 6). In the *in vitro *infection model, the integrated HIV-1 DNA level decreased from 1,873,330 copies/10^7 ^resting CD4^+ ^T cells without preincubation to 173,501 copies/10^7 ^resting CD4^+ ^T cells with two-days preincubation. For patients' nos. 4, 13, and 14, the levels of HIV-1 integrated DNA copies were 1,190 ; 13,100 and 4,100 copies/10^7 ^resting CD4^+ ^T lymphocytes with one-day preincubation and 370 ; <100 and 1,700 HIV-1 integrated DNA copies/10^7 ^resting CD4^+ ^T lymphocytes with two-days preincubation, respectively. These results confirmed that the decrease of HIV-1-Ag-SCs observed with preincubated cells was due to unintegrated HIV-1 DNA decay (Fig. 3A and 3B).

## Discussion

Detection and enumeration of resting CD4^+ ^T lymphocytes latently infected with HIV-1 is important to quantify cellular HIV-1 reservoir and to anticipate HIV-1 reservoir modifications that may result from new antiretroviral therapies. In this point context, understanding mechanisms by which reservoirs of HIV-1 latently infected cells are established and maintained *in vivo *is crucial. The preintegration phase of latency has been reported in viremic patients [12,23]. However, the biological activity of this reservoir comprised of resting CD4^+ ^T lymphocytes harboring unintegrated HIV-1 DNA has not been observed. So, by using a proof-of-concept model of an *in vitro *HIV-1 latent infection to valid the experimental protocol, we proposed to explore the functional preintegration reservoir and its capacity to induce a rescuable virus production in untreated and HAART-treated patients.

Our approach permitted to enumerate HIV-1-SCs and to assess the functionality of unintegrated HIV-1 DNA. The capacity of this potential reservoir to produce viral proteins cannot be directly observed because resting CD4^+ ^T lymphocytes harbor unintegrated or integrated HIV-1 DNA before cell polyclonal activation. When resting CD4^+ ^T cells are activated *in vitro*, at least a part of extrachromosomal viral DNA is integrated into the host cell genome and generates rescuable virus production that defines the inducible functional HIV-1 preintegration reservoir which can not be distinguished from the total functional HIV-1 reservoir. However, the addition of an HIV-1 integrase inhibitor that inhibits the HIV-1 DNA integration into the host genome allows the enumeration of CD4^+ ^T cells harboring integrated HIV-1 DNA able to enter a replicative cycle.

We first demonstrated in an *in vitro *HIV-1 latent infection model that HIV-1 production was rescued from infected resting CD4^+ ^T lymphocytes after polyclonal activation. This observation was extended by showing that addition of the HIV-1 integrase inhibitor L-731,988 in culture medium efficiently prevented HIV-1 production from stimulated CD4^+ ^T lymphocytes. Moreover, preincubation of infected resting CD4^+ ^T cells in the absence of activating stimuli for 1 and 2 days led to the decline of the number of HIV-1-Ag-SCs indicating a strong decay of unintegrated HIV-1 DNA over time. Thus, these approaches allowed us to assess the functionality and lability of the HIV-1 reservoir in the preintegration phase of latency in resting CD4^+ ^T lymphocytes as well as the role of unintegrated HIV-1 DNA in rescuable virus production. In agreement with previous reports [7,12,15], the *in vitro *latent infection of resting CD4^+ ^T lymphocytes generated a pool of infected cells in the preintegration phase of HIV-1 latency able to integrate some extrachromosomal HIV-1 DNA forms into their genome after polyclonal stimulation.

In untreated patients, we explored the functional preintegration reservoir and its capacity to induce rescuable viral production. We first observed a significant decline of the number of HIV-1-Ag-SCs when purified resting CD4^+ ^T lymphocytes were polyclonally activated and cultured with HIV-1 integrase inhibitor, highlighting the presence of a circulating inducible and functional preintegration HIV-1 reservoir in all of these patients. As suggested by the decrease of rescuable viral production when resting CD4^+ ^T cells were preincubated before their polyclonal activation, this reservoir was labile. These results are in agreement with those observed with the *in vitro *experimental model of HIV-1 latent infection and with data reporting that unintegrated HIV-1 DNA is the most common form of latent virus in resting CD4^+ ^T lymphocytes from untreated patients [12,23]. In untreated patients, the *de novo *infection of resting CD4^+ ^T cells is insured by the HIV-1 production from activated infected CD4^+ ^T cells, which leads to the continual replenishing of the pool of infected resting CD4^+ ^T lymphocytes harboring unintegrated HIV-1 DNA.

In sustained responder to HAART, the results obtained using the HIV-1 integrase inhibitor demonstrated that the inducible functional preintegration reservoir was present in some individuals. As observed in the model of latent infection and in untreated patients, this reservoir was functional and labile. These results provide strong evidence for a contribution of the residual viral replication in the HIV-1 reservoir replenishment despite sustained response to HAART.

The characterization of a functional preintegration reservoir and of spontaneous HIV-1-producing CD4^+ ^T lymphocytes in untreated patients and in sustained responders to HAART could provide a means for determining the mechanisms of the viral persistence. In untreated patients, the viral production is insured by activated infected CD4^+ ^T lymphocytes and by a pool of HIV-1-infected resting CD4^+ ^T cells that spontaneously produce viral particles with neither expression of phenotypical activation markers nor presence of exogenous activation stimuli [10,16,17]. HIV-1 infection induces aberrant immune activation of latently infected CD4^+ ^T cells associated with an enhancement of expression of certain host genes despite the absence of expression of classical cell-surface activation markers [16]. In sustained responders to HAART, resting CD4^+ ^T lymphocytes do not spontaneously release HIV-1 [16,17]. However, the latent HIV-1 persistence could be insured by the intrinsic stability of the HIV-1 reservoir and by the presence of spontaneously activated CD4^+ ^T cells despite efficient antiretroviral treatment, as previously suggested by other reports [1,17]. Reactivation of latently infected resting CD4^+ ^T cells, probably resulting from immunological responses to specific antigens or induction by cytokines, leads to the release of virus able to infect neighbouring resting or activated CD4^+ ^T cells [17]. To address this issue, we assessed the presence of the spontaneous HIV-1-producing CD4^+ ^T lymphocytes in the peripheral blood of untreated and sustained responder HAART-treated patients. As expected, spontaneous HIV-1-Ag-SCs were detected in untreated patients but also in sustained responders harboring a functional preintegration reservoir. These data suggest that the preintegration reservoir in HAART-treated patients could be replenished via *de novo *infection of resting CD4^+ ^T cells by HIV-1 virions released from spontaneously activated CD4^+ ^T lymphocytes.

## Conclusion

Taken together, all these data suggest that different mechanisms such as the residual viral replication and the HIV-1 latent reservoir reactivation are responsible for the HIV-1 persistence. Despite the highly efficiency of HAART, the detection of a functional preintegration reservoir associated to the presence of spontaneously activated infected CD4^+ ^T lymphocytes is in favour of a continual replenishment of the latent HIV-1 reservoir *in vivo*. This observation highlights the need for a complete suppression of viral replication in addition to HIV-1 cure by treatments aimed at inhibiting integration of HIV-1 extra-chromosomal DNA and preventing from establishment of the proviral HIV-1 reservoir.

## Methods

### In vitro model of latently infected resting CD4^+ ^T cells

We designed a model of latent HIV-1 infection to obtain resting CD4^+ ^T lymphocytes harboring unintegrated viral genomes. For this purpose, peripheral blood mononuclear cells (PBMC) obtained from healthy donors were isolated by Ficoll-Hypaque density gradient centrifugation. Unstimulated cells were exposed to 1 × 10^2 ^TCID_50 _of HIV-1 strain NL_4-3 _for 30 min at 4°C, extensively washed to remove unbound virions and subsequently incubated for 24 h at 37°C in 5% CO_2 _[24]. Infected PBMC were then washed 5 times and cryopreserved in liquid nitrogen until use. Resting CD4^+ ^T lymphocytes were isolated from infected PBMC using a Rosette Sep™ CD4 cell enrichment cocktail including antibodies (Abs) directed against CD8, CD16, CD19, CD36, and CD56 according to the manufacturer's instructions (Stemcell Technologies, Meylan, France), and a Custom Cocktail containing Abs directed against HLA-DR, CD69, and CD25 cell receptors (Stemcell Technologies, Meylan, France) to deplete spontaneously activated CD4^+ ^T cells.

### Patients

Twelve untreated and eleven HAART-treated patients were recruited after written informed consent. Patients' characteristics and treatments are presented in Table 1. Plasma viral load was measured by a real-time HIV-1 RNA PCR assay (Cobas AmpliPrep/Cobas TaqMan HIV-1 assay; Roche Diagnostics Systems, Meylan, France). The CD4^+ ^T cell count was determined by flow cytometry (FC500; Beckman-Coulter, Villepinte, France) after cell staining with fluorescein isiothiocyanate (FITC), rhodamine 1 (RD1), energy coupled dye (ECD), and phycoerythrin-cyanine 5 (PC5)-conjugated Abs directed against the CD45, CD4, CD8 and CD3 receptors, respectively (Cyto-Stat^®^/tetraChrome™, Beckman-Coulter).

### Isolation of CD4^+ ^T lymphocytes

CD4^+ ^T cells were purified from 15 ml of EDTA-treated blood samples using the Rosette Sep™ CD4 cell enrichment cocktail, according to the manufacturer's instructions (Stemcell Technologies) without depletion of spontaneously activated CD4^+ ^T lymphocytes. From 0.6 to 2 × 10^6 ^CD4^+ ^T lymphocytes (median 1.08 × 10^6^) were stored in liquid nitrogen.

### Isolation of resting CD4^+ ^T cells

Resting CD4^+ ^T cells from HIV-1-infected patients were purified from 20 ml of EDTA-treated blood samples using the Rosette Sep™ CD4 cell enrichment cocktail, according to the manufacturer's instructions (Stemcell Technologies). Spontaneously activated CD4^+ ^T cells were depleted using a Custom Cocktail containing Abs directed against HLA-DR, CD69, and CD25 membrane receptors (Stemcell Technologies). As controlled by FACS, the enriched CD4^+ ^T cell population contained more than 99% of resting CD4^+ ^T cells. Aliquots from 0.8 to 7.3 × 10^6 ^resting CD4^+ ^T cells (median 2.51 × 10^6^) were stored in liquid nitrogen.

### CD4^+ ^T cells activation

Thawed resting CD4^+ ^T cells were cultured in flasks at the concentration of 1 × 10^6 ^cells/ml and stimulated with monoclonal human Abs directed against CD3 and CD28 receptors plus mitomycin-treated CD8^+ ^T cell-depleted PBMC from HIV-1-seronegative individuals. Briefly, 24-well culture plates (Falcon, Meylan, France) were coated overnight with anti-CD3 Abs at the final concentration of 2 μg/ml. PBMC from controls were depleted of CD8^+ ^T cells using Human CD8 cell Depletion Cocktail (Stemcell Technologies), according to the manufacturer's instructions and then treated with mitomycin (25 μg/5 × 10^6 ^CD8^+ ^T cell-depleted PBMC, 30 min at 37°C under gentle agitation). After washings with phosphate-buffered salt pH 7.2 (PBS), enriched CD4^+ ^T cells were cultured with 3 × 10^6 ^mitomycin-treated CD8^+ ^T cell-depleted PBMC plus soluble anti-CD28 Abs at the final concentration of 2 μg/ml. To prevent infection of neighboring cells by *de novo*-synthesized HIV-1, 1 μg/ml of the HIV-1 entry inhibitor T20 (enfuvirtide; Roche Pharma, Nutley, N.J.) was added in culture medium as previously described [20,21]. These culture conditions have been previously shown to induce stimulation of more than 98% of resting CD4^+ ^T cells [21]. Cells were cultured at 37°C in a 5% CO_2_-humidified atmosphere and tested at day 5 using the HIV-1-Ag-ELISpot assay. In addition, unstimulated CD4^+ ^T cells not exposed to anti-CD3 Abs, anti-CD28 Abs, and mitomycin-treated CD8^+ ^T cell-depleted PBMC were cultured under the same conditions and generated <1 to 5 HIV-1-Ag-SCs/10^7 ^resting CD4^+ ^T lymphocytes.

### Exploration of the preintegration reservoir

To study the mobilization of the preintegration reservoir, we compared the HIV-1-antigens production from resting CD4^+ ^T cells that were activated and cultured with or without the HIV-1 integrase inhibitor L-731,988 kindly provided by Merck Sharp & Dohme-Chibert (Paris, France) at the final concentration of 40 μM (Fig. 1A) as previously described by Zhou *et al. *[15]. In addition, to assess the correlation between the unintegrated HIV-1 DNA decay in cell cultures and the decline of rescuable viral production, resting CD4^+ ^T cells were preincubated in culture medium without activation for 1 and 2 days before polyclonal stimulation (Fig. 1B). This preincubation time in the absence of activating stimuli could allow for the decay of unintegrated HIV-1 DNA.

### HIV-1-Ag-ELISpot assay

Immobilon-P membrane 96-well plates (MAIPN 4550; Millipore Corporation, Bedford, Mass.) were coated overnight at 4°C with a mixture of anti-HIV-1 polyclonal Abs prepared as previously described [21]. Sera from 10 HIV-1 patients with a complete HIV-1-Ab-specific serologic pattern in Western blot were pooled, adsorbed on CEM cells at a concentration of 5 × 10^6 ^cells/ml for 60 min at 37°C under agitation, and used at 1:250 dilution. After three washings with PBS, 1 × 10^5 ^cultured CD4^+ ^T lymphocytes were seeded into each well. Plates were incubated for 24 h at 37°C in a 5% CO_2_-humidified atmosphere. After nine washings (3 × PBS, 3 × PBS-0.05% Tween_20_, 3 × PBS), 100 μl of biotinylated anti-p24 monoclonal Ab at 1:1,000 dilution (Genetics systems HIV-1 Ag EIA; Bio-Rad, Marnes la Coquette, France) were added and incubated for 6 h at 37°C. After three PBS washings, a solution of alkaline phosphatase-labeled streptavidin diluted at 1:1,000 in PBS was added and plates were incubated 45 min at 37°C, washed three times in PBS and developed with a chromogenic substrate (a mixture of 5-bromo-4-chloro-3-indolyl phosphate and nitroblue tetrazolium; Sigma, St. Louis, Mo.). Immunospots appeared as purple precipitates after 10 min and were counted by video camera imaging and computer-assisted analysis (KS ELISPOT; Carl Zeiss Vision, Hallbermoos, Germany). When HIV-1-Ag-SCs were undetectable, results were expressed as <1 HIV-1-Ag-SCs/10^7 ^resting CD4^+ ^T lymphocytes according to the number of tested cells.

Spontaneously HIV-1-Ag-producing CD4^+ ^T lymphocytes were also enumerated. Briefly, 1 × 10^5 ^purified CD4^+ ^T lymphocytes not depleted for HLA-DR^+ ^CD69^+ ^CD25^+ ^cells were directly seeded into each well of ELISpot plates, cultured 24 h without polyclonal stimuli, and HIV-1-Ag-SCs were detected using the ELISpot assay described above.

### Flow cytometric analysis

The viability of CD4^+ ^T lymphocytes was analyzed at 1 and 2 days of cell pre-culture and at the end of cell stimulation. Gate were set on lymphocytes based on Forward-Scatter vs Side-Scatter histogram and CD4^+ ^T lymphocytes were defined in the corresponding monoparametric histograms CD4-FITC. CD4^+ ^T cells activation was assessed by the expression of the activation marker CD69 using anti-CD69-conjugated-phycoerythrin (PE) Abs. Disrupted membranes of dead cells allow for the fluorescent 7-amino-actinomycin D (7AAD) internalization and nuclear DNA binding, and viable cells were defined as the percentage of 7AAD negative events in the monoparametric histogram 7AAD (all reagents from Beckman-Coulter).

### Integrated HIV-1 DNA real-time PCR assays

*In vitro *latently infected CD4^+ ^T lymphocytes and CD4^+ ^T cells from untreated and treated patients were recovered after ELISpot assays in order to estimate the level of unintegrated HIV-1 DNA by PCR experiments. Total DNA was extracted using the QIAamp DNA blood Midikit (Qiagen; Hilden, Germany) according to the manufacturer's instructions and stored at -80°C. Integrated HIV-1 DNA was then detected using *Alu*-LTR-based real-time nested-PCR procedure according to Brussel *et al*. [25], with the following modifications. The LTR-targeted region was amplified by PCR and then sequenced for each patient to compare LTR and primers L-M667 and AA55M sequences. DNA from 6 out of 9 patients had perfect matches for the two primers and quantification was carried on. The first amplification with primers L-M667 only (control) or with Alu1 and Alu2 (integrated) had an annealing temperature of 65°C. To reduce unspecific background, 2 μl of the first amplification was digested with 20 U of Exonuclease I (New England Biolabs GmbH; Frankfurt, Germany) in 20 μl for 2 h at 37°C. The nuclease was heat inactivated at 80°C for 20 min, and 2 μl of the digestion was amplified at 65°C with primers Lambda T and AA55M in presence of SYBR Green. HIV-1 proviral DNA was normalized to cell number by quantitating cellular CCR5 gene. Primers sequence for CCR5 gene amplification were [5'-GTGAAGCAAATCGCAGCCCGC-3'] and [5'-GCAGCATAGTGAGCCCAGAAG-3']. The detection threshold of integrated HIV-1 DNA using the *Alu*-LTR real time PCR is 100 integrated HIV-1 DNA copies/10^7 ^tested cells.

### Statistical analysis

Comparison of results was done by Wilcoxon signed-rank test. A *P *value < 0.05 was considered as statistically significant.

## Abbreviations

HAART- highly active antiretroviral therapy.

HIV-1-Ag-ELISpot- HIV-1-antigens-ELISpot assay.

HIV-1-Ag-SCs- HIV-1-antigens-secreting cells.

HIV-1-SCs- HIV-1-secretring cells.

## Competing interests

The author(s) declare that they have no competing interests.

## Authors' contributions

GP and JPV designed research. GP, ET, CM and MS performed research. GP, YAT, ET, CM, MS, VB, JR and JVP analyzed data. GP, YAT, CM, MS and JPV wrote the paper.
